# A Call for an Inclusive Path Forward: Developing a Roadmap for Diversity and Equity for Field Epidemiologists

**DOI:** 10.1002/puh2.70152

**Published:** 2025-10-21

**Authors:** Rachel Hammersley‐Mather, Tambri Housen, Sonia Palmieri, Emma Field

**Affiliations:** ^1^ National Centre for Epidemiology and Population Health Australian National University Canberra Australia; ^2^ School of Medicine and Public Health University of Newcastle Callaghan Australia; ^3^ Department of Pacific Affairs Australian National University Canberra Australia

**Keywords:** diversity, equity and inclusion, field epidemiology, women in global health

## Abstract

Originating in 1951 in the United States with a cohort of male physicians, field epidemiology training programs (FETPs) now support workforce development in more than 200 countries and territories, with graduates of diverse genders and professional backgrounds. In 2018 a group of leading field epidemiologists identified avenues to modernize the field, published in *The Path Forward: The Global Field Epidemiology Roadmap*. The roadmap lacked intersection with contemporary concerns, including diversity, equity, and inclusion (DEI). Mirroring global health workforce trends that see women overrepresented at the frontline and underrepresented across leadership, a review of the processes to develop The *Path Forward* highlights inequities across field epidemiology leadership. With the nascent *Global Field Epidemiology Partnership* publishing a strategic plan acknowledging DEI, we provide recommendations to understand the modern population of field epidemiologists, create space for untraditional voices, and diversify leadership.

## 1 Introduction

Like health itself, the global health workforce is characterized by inequalities, including that women represent 70% of the frontline workforce yet occupy fewer than 25% of leadership roles [1]. The COVID‐19 pandemic reinforced gender inequities within the global health workforce but also illuminated the contributions of a specific kind of health worker: the epidemiologist [2]. Amongst other things, field epidemiologists or “disease detectives” work with public health surveillance data to identify outbreaks, support response, and provide data for evidence‐based decision‐making. Amid calls for broader investment in epidemiological capability in the post‐pandemic landscape, the *Global Field Epidemiology Partnership* (GFEP) was launched in October 2023 with the vision that “all countries have robust field epidemiology capacities to protect their population's health and contribute to global health security.” [3]. A joint initiative of the United States (US) Centers for Disease Control and Prevention (CDC), the Training Programs in Epidemiology and Public Health Interventions Network (TEPHINET), the World Health Organization (WHO), and “other stakeholders”, GFEP's strategic plan proclaims it adheres to the WHO values of diversity, equity, and inclusion (DEI), while purposefully seeking balanced gender representation [3]. With the strategy finalized in the months after Trump returned to the White House, it now exists in distinct opposition to his commitment to eliminate “discriminatory DEI practices” [4]. The GFEP Steering Committee must deeply consider how to walk the DEI talk at a time when US Government documents are banned from even using the words the acronym comprises [5]. Here we reflect on lessons learned from previous global field epidemiology strategies. We focus on equality for women to highlight key opportunities to mainstream DEI priorities across all levels of field epidemiology.

## 2 A Brief Summary of Field Epidemiology

Although COVID‐19 shone a spotlight on the role of epidemiologists in national, regional, and global health, the field epidemiology community is well established. As distinct from clinical or academic epidemiology, field epidemiologists practice epidemiology in “real time and real place,” identifying and responding to urgent public health problems, such as outbreaks, through contributing to and appraising public health surveillance data [6]. The model that characterizes field epidemiology training evolved from the CDC Epidemic Intelligence Service (EIS) program, which enrolled its first cohort in 1951 [6]. Comprising 23 men—22 physicians and an engineer—the cohort was recruited under the rationale of biodefense in the face of looming biological warfare [7]. The model became international in 1975 when the Canadian field epidemiology training program (FETP) was established and left North America in 1980 with the commencement of FETP Thailand [7]. As of 2023, there were 98 FETPs training public health workers across more than 200 countries and territories [8, 9]. Although programs are ostensibly contextualized to meet country needs, there is no specific guidance on how to mainstream an FETP to align with, for example, cultural priorities for learning [10]. This has meant that although countries replicated the US program, copying “core learning principles, process, and activities,” how they have adapted to local cultures, contexts, and needs is not well understood. Similarly, although the program was initially designed for a cohort of White males and has expanded globally to include all genders and a spectrum of professionals, there are no published gender analyses of FETPs exploring the differential impacts the training model has on women [7, 10]. As its origins in the US, the profile of field epidemiologists has fundamentally changed. Although there are no data on the demographic composition of the global field epidemiology workforce, a recent global online survey focused on emergency response collected demographic data on field epidemiologists [11]. Coming from 64 countries, slightly more than half of respondents were women, the median age was 39, and almost three‐quarters were graduates of an FETP. Less than a quarter of respondents had a background in medicine, whereas more than half had a technical background in public health. Although there were limitations to the data collected by the survey—including online delivery and a small sample size (*n* = 282)—it nonetheless highlights the significant evolution of field epidemiologists from the 1950s to the present day.

## 3 A Roadmap to Modernity: Developing the Field Epidemiology Global Strategy

Following informal collaborations between FETP directors who sought to share learning and resources, TEPHINET was established in 1997 through support from the WHO, the CDC, and the Fondation Merieux [8]. TEPHINET is the global network for FETPs, overseeing the quality of FETPs and seeking to foster best practices across them. Today, it is primarily funded by the CDC. In its November 2018 strategy document *The Path Forward: The Global Field Epidemiology Roadmap*, TEPHINET articulated an agenda to “modernize FETPs” through implementing eight high‐level recommendations with oversight from a Strategic Leadership Group (SLG) [12].

Borne out of a 5‐day meeting at the Rockefeller Foundation Bellagio Center in Italy in June 2018, the strategy neither reflected the modern profile of the field epidemiology community, nor addressed modern DEI priorities. We suggest that these omissions are at least in part attributable to a perceptible gender imbalance among meeting attendees.

To evaluate gender representation at this and subsequent meetings, we reviewed lists of participants to identify perceived gender identities. We developed a hierarchy to determine gender: First, we identified the gender identities of participants who were personally known to one or more authors. Next, we identified participants by gendered title (e.g., Ms., Mr., Mrs., and Mx.). If no title was available in a report, we used the Google search engine to determine the gender identity of participants by title or gender pronouns (e.g., she/her, they/them, and he/his). We acknowledge the limitations of our methods, including opportunities to incorrectly perceive gender identities. However, given the small, familiar community reviewed, we are confident in our assessments.

Of the 19 participants listed in *The Path Forward: The Global Field Epidemiology Roadmap* report, women represent less than a third of participants (see Table 1). Gender representation is also unbalanced in the report authorship—no females were credited with authorship [12].

**TABLE 1:** Gender representation of participants within key strategic meetings and groups concerning the development and implementation of *The Path Forward: The Global Field Epidemiology Roadmap* [12, 13, 14].

Meetings and groups	Female (%)	Male (%)	Total
Roadmap meeting	6 (31.6)	13 (68.4)	19
Roadmap meeting authors	0 (0)	4 (100)	4
Implementation meeting	3 (14.3)	18 (85.7)	21
Implementation meeting planning committee	0 (0)	5 (100)	5
Implementation meeting author	0 (0)	1 (100)	1
Strategic Leadership Group	4 (26.7)	11 (73.3)	15
Working groups—total participants	21 (50)	21 (50)	42
Working groups—chairs	2 (25)	6 (75)	8

To develop an implementation plan for the Roadmap, a 3‐day meeting was held in Geneva, Switzerland in February 2019; the subsequent report is called *A Report of the Global Field Epidemiology Roadmap Implementation Meeting* [13]. The Meeting Planning Committee again suffered from gender imbalance. The report lists 21 meeting participants, of whom less than 15% were female, plus one female rapporteur. One of the female participants is listed as the technical aid of another (male) participant.

## 4 Overseeing the Roadmap: Implementing the Global Field Epidemiology Strategy

*The Path Forward: The Global Field Epidemiology Roadmap* outlines eight key recommendations, the first being to establish a “broadly representative” SLG [12]. Exactly who the SLG are representing is not defined by the roadmap or implementation report, but it can be assumed that the group was not intended to be representative of the global community. A session on the SLG and the roadmap at the 11th TEPHINET Global Scientific Conference, held in Panama in September 2022, described the SLG composition as “high‐level, influential, broadly experienced global health leaders” with a mission to “provide high‐level driving force for progress.” [14]. Echoing wider global health leadership trends, the composition of the SLG would suggest there are fewer “high‐level” women in field epidemiology, as women represent only a little over a quarter of the SLG.

Each of the eight recommendations had a corresponding working group, a quarter of which were chaired by women (see Table 1). Women's contributions to groups varied: More than two‐thirds of the group charged to monitor required improvements and changes to the FETP Enterprise (*Recommendation 2*) were women, whereas there were none in a group tasked with assuring sustainable funding (*Recommendation 7*) [14]. Although women comprised almost half of all individuals supporting working groups, with some individuals contributing to multiple groups, their total mean and median representation was 39%. Although still under parity, that women's contributions increased substantially in the *working* groups is reflective of the broader global health trends, where women are overrepresented in the frontlines and severely underrepresented in leadership.

## 5 An Inclusive Path Forward

Designated by TEPHINET, the theme of the 2023 World Field Epidemiology Day was “Increasing Diversity, Equity, and Inclusion in Field Epidemiology.” As the new lead in developing global field epidemiology strategy, GFEP will do well to consider how and where they can lead by example. With post‐pandemic calls for further investment in field epidemiology training, we put forward foundational recommendations for field epidemiology leadership to consider how to improve DEI as it charts a new path forward. These are relevant whether implementing a local FETP, coordinating a regional network or representing the field epidemiology community at the global level, and can be contextualized to be appropriate given the conversation and priorities for gender equality within a given space.

### 5.1 Recommendation 1: Understand Your Diverse Population

The profile of the modern field epidemiologist is unclear. For a field that focuses on collecting and analyzing population data, it is remarkable that systematic data of FETP enrolments, and graduates have not been routinely collected or published by global coordinating bodies like TEPHINET, including on demographics and how training experiences intersect with culture, context and gender, and other aspects of inclusion. Further understanding of the diversity of FETPs and field epidemiologists—including through gender analyses of FETPs that consider intersectional forms of representation—will highlight diverse experiences in all aspects of field epidemiology, including in leadership and decision‐making. It will also allow epidemiologists to do what they are supposed to do best: compare data to make evidence‐based decisions. The data will reveal different gendered stories in different countries and regions. These can then be compared so that solutions can be tailored for specific FETPs and regional networks given the dynamics between gendered challenges and priorities for gender equality within a given cultural context.

### 5.2 Recommendation 2: Make Space

A global roadmap requires global perspectives. Strategy documents must be underpinned by the priorities of people with diverse experiences and expertise, and from organizations outside key funding bodies and countries. The criteria for being offered a seat at the meeting table needs to extend beyond being an expert epidemiologist. Inclusion must be intersectoral and intersectional, with active participation sought from experts in gender, disability, and social inclusion. For example, the “Regional Roadmap to Advance Field Epidemiology Capacities in the WHO South‐East Asia Region 2025–2029” did not consult gender equality experts in its formation, and it shows: despite being a WHO publication underpinned by WHO commitments, it does not address gender considerations in either broad or specific terms [15]. Inclusion must start with diversity in Meeting Planning Committees, go through to authoring reports, and extend to every level of decision‐making across the field epidemiology community and priorities: from governance to finance and everything in between.

### 5.3 Recommendation 3: Diversify the Leadership

Until the appointment of the GFEP Steering Committee, we suggest that women in field epidemiology are even worse off than their colleagues in other areas of global health, occupying less than a quarter of key leadership positions. We welcome the culture diversity of the 12‐member GFEP Steering Committee, and its near gender parity, with five women (41.7%) [16]. Still, linking back to our first recommendation, we think it is imperative that GFEP's leadership reflects the field epidemiology frontline. The data collected from Recommendation 1 should be used to inform leadership targets within each institution—not just in terms of gender, but other aspects of representation, including region, language, education, and disability. The benefits of more inclusive leadership are well expounded and extend far beyond arguments for equality across the health workforce; fundamentally, inclusive leadership benefits the communities that health workers serve [2, 16, 17, 18]. At the level of individual FETPs, it is essential that trainees can see their intersectional identities within the program staff and faculty, including investment in trainers to ensure workshops can be delivered without fly‐in–fly‐out epidemiologists, and that mentors are appropriately matched given cultural norms on gender.

As Wenham and Davies observed in 2022, “WHO has neglected to mainstream gender in the policies and practices that it promotes for the prevention and detection of, and response to, infectious disease outbreaks.” [2]. So has field epidemiology leadership. To increase equity and inclusion, it is imperative that the path forward is led by field epidemiologists, in all their diversity.

## Author Contributions

RHM conceived of the paper, conducted the analysis, and drafted and approved the final manuscript. EF supported the analysis, made technical contributions to the paper, and read and approved the final manuscript. TH and SP made technical contributions to the paper, and read and approved the final manuscript.

## Funding

The authors have nothing to report.

## Ethics Statement

The authors have nothing to report.

## Consent

The authors have nothing to report.

## Conflicts of Interest

The following section has been drafted using the subheadings on an ICMJE disclosure form. Rachel Hammersley‐Mather, Tambri Housen, and Sonia Palmieri all work for programs that receive funding from the Australian Government's Department of Foreign Affairs and Trade. Tambri Housen additionally supports a program funded by Global Fund. In the past 48 months, Tambri Housen has been contracted by Sydney University and Aspen Medical in technical advisory roles, for which she has received consulting fees. Further, Tambri Housen has been contracted and received contracting fees, including travel support, from the Training Programs in Epidemiology and Public Health Interventions Network (TEPHINET) to support the development of the revised Global Field Epidemiology Strategy. Tambri Housen does not receive payment but is The Western Pacific Regional representative on the World Health Organization (WHO) Academy Quality Committee; a member of the Global Outbreak Alert and Response Network (GOARN) capacity building and training network; a member of the FETP (Field Epidemiology Training Programs) Learning Advisory Council; and was a Member of TEPHINET's Strategic Leadership Group Working Group 2. Emma Field is the director of the Australian Field Epidemiology Training Program (the Master of Philosophy in Applied Epidemiology at Australian National University) and a member of the Global Field Epidemiology Partnership Technical Working Group on Field Epidemiology Competencies.

## Data Availability

The authors confirm that the data supporting the findings of this study are available within the article through the sources that are referenced and hyperlinked.
